# Chinese-Language Montreal Cognitive Assessment for Cantonese or Mandarin Speakers: Age, Education, and Gender Effects

**DOI:** 10.1155/2012/204623

**Published:** 2012-07-09

**Authors:** Ling Zheng, Evelyn L. Teng, Rohit Varma, Wendy J. Mack, Dan Mungas, Po H. Lu, Helena C. Chui

**Affiliations:** ^1^Department of Neurology, Keck School of Medicine, University of Southern California, Los Angeles, CA 90033, USA; ^2^Department of Ophthalmology, Keck School of Medicine, University of Southern California, Los Angeles, CA 90033, USA; ^3^Department of Preventive Medicine, Keck School of Medicine, University of Southern California, Los Angeles, CA 90033, USA; ^4^Department of Neurology, School of Medicine, University of California, Davis, Sacramento, CA 95817, USA; ^5^Department of Neurology, David Geffen School of Medicine, University of California, Los Angeles, CA 90095, USA

## Abstract

The Montreal Cognitive Assessment Chinese-Language Los Angeles version (MoCA-ChLA) was developed and administered during an in-home interview to 1,192 participants (mean age 62.5 years, mean education 11.6 years) in a population-based Chinese American Eye Study (CHES) in Los Angeles. The MoCA-ChLA score (mean ± SD) was 23.8 ± 4.2 with little ceiling and no floor effects. The score increased with higher education, decreased with advancing age, and was not related to gender. Compared to the education 1–6 years group, the mean MoCA-ChLA score was 2.6 and 4.6 higher in the education 7–11 and 12–20 years groups, respectively. The Mandarin- (*n* = 612) and Cantonese- (*n* = 612) speaking subgroups performed comparably; Cronbach's alpha of the MoCA-ChLA score was 0.78 and 0.79 for these two groups, respectively. Item response theory analysis showed good discriminating power for executive function and memory. These properties support the MoCA-ChLA as a useful screening tool for aging and dementia studies for Mandarin or Cantonese speakers.

## 1. Introduction

The Chinese American population is one of the most rapidly growing minorities in the United States [1, 2]. According to the USA Bureau of the Census (2010), the number of Chinese Americans increased 229% from 1.62 to 3.8 million from 1990 to 2010 [2]. One-third of Chinese Americans (1.25 million) reside in California, and the number of Chinese Americans with dementia is expected to more than triple in the next 30 years [3]. However, few studies have focused on the screening for cognitive impairment among Chinese-Americans. Linguistically and culturally appropriate cognitive screening tests to detect and stage cognitive impairment are needed to facilitate early detection and intervention. The lack of such instruments also limits the participation of Chinese Americans in cognitive and aging research.

 The Montreal Cognitive Assessment (MoCA) is a brief cognitive screening test designed to distinguish individuals with mild cognitive impairment (MCI) who perform in the normal range of the Mini-Mental State Examination (MMSE) from cognitively normal elderly [4]. Since Chinese Americans migrated primarily from Mainland China, Hong Kong, Macau, and Taiwan, the Chinese-speaking population in the USA consists of three major cultural subgroups whose primary dialect is Mandarin [Putonghua], Cantonese, or Taiwanese [5]. These three groups may use different words or expressions for some concepts and may differ in level of education.

We therefore translated and adapted the MoCA for Mandarin-, Cantonese-, and Taiwanese-speaking Chinese Americans with one common written version named the MoCA Chinese Los Angeles version (MoCA-ChLA). The MoCA-ChLA was administered to 1,192 Chinese residents in Monterey Park, California, as part of a population-based study. The City of Monterey Park in Los Angeles County, the first suburban Chinatown in the USA [6], has the highest percentage of Chinese Americans of any USA municipality, at 43.7% of its population (60,269) in 2010 [2]. The goal of this study was to characterize the effects of preferred language, age, education, and gender on the performance of the MoCA-ChLA in this minority population.

## 2. Methods

### 2.1. Contents of the MoCA

The MoCA was specifically developed as a screening tool for mild cognitive impairment and early dementia [4]. Compared to the MMSE, it includes more memory items and assesses key aspects of executive function. The MoCA has 12 items that assess 6 cognitive domains including short-term memory, visuospatial construction, executive functions, attention and concentration, language, and temporal and spatial orientation (Table 1). The MoCA is scored on a 30-point scale and takes about 10 minutes to administer and 1 minute to score. In the original study, the MoCA was administered to 277 English- or French-speaking Canadians, aged 65 years or older, with mean education of 11.9 years (averaged over normal, MCI, and Dementia groups). One point was added for persons with education of less than 12 years. In terms of identifying MCI, a MoCA score of ≤26 showed a sensitivity of 90% and a specificity of 87% in the original study [4]. Later, a study in the UK found sensitivity of 83% and much lower specificity of 50% [7]; a Korean study reported a sensitivity of 89% and a specificity of 84% [8]; a recent study in Japan reported a sensitivity of 93% and a specificity of 87% in screening for MCI [9]. These studies all added 1 point for education <12 years with the exception of the Korean study, where 1 point adjustment was made for education <6 years.

### 2.2. Development of MoCA-ChLA

As of April 2010, the MoCA has been translated independently into Mandarin, Cantonese, and Taiwanese versions (http://www.mocatest.org/). We carefully reviewed the instrument and instruction manual of the original MoCA in English, as well as the three Chinese versions developed separately in Beijing, Hong Kong, and Taiwan. The written words in the three Chinese versions are not identical and in some instances misinterpreted the intention of the original MoCA test item. For example, in the Hong Kong version, sentence repetition seems to assess articulation more than attention, as the subject is asked to repeat something like “Sasha age sixty six” [10]. In addition, all 3 Chinese versions tend to follow a literal translation of the English instructions, such that the resulting Chinese sentences sound unnatural and are difficult to comprehend, especially to individuals with little or no formal education.

 In developing the MoCA-ChLA, we attempted to maintain the neuropsychological intention of the original MoCA instrument. We incorporated appropriate parts of the existing Chinese versions, made modifications of other parts, and ensured that the spoken instructions would be easily understood by Mandarin, Cantonese, and Taiwanese-speakers. In the 5-word learning test, we changed “face” to “hair” and “velvet” to “teacup.” “Face” is a single character word in Chinese that could mean the physical face or the social face. “Velvet” can be either a 2-character or a 3-character word depending on the dialect, and could be unfamiliar to older and low-educated Chinese. In contrast, the words “hair” and “teacup,” like “church”, “daisy,” and “red” are all unambiguous 2-character words in Chinese and familiar to all. We changed the phonemic fluency task (generating words beginning with letter F within 60 seconds) to a semantic fluency task (generating names of 4-legged animals) because Chinese is a monosyllabic language and each phoneme has multiple ambiguous meanings, which undermines the meaningfulness of phonemic fluency in Chinese. Generating animal names has shown good validity as a measure of verbal fluency among Chinese elderly [11, 12].

The development of the MoCA-ChLA was an iterative process. First, the MoCA-ChLA items were adapted jointly by a bilingual neuropsychologist (ELT) and a bilingual epidemiologist (LZ). Subsequently, they were back translated into English by a bilingual psychologist (PL) without knowledge of the original MoCA items. Successive versions were reviewed for clarity by focus groups of native Mandarin, Cantonese, or Taiwanese speakers in the lay community. Wording was adjusted where necessary, and the process was repeated until the final MoCA-ChLA was considered by the two bilingual psychologists (ELT and PL) to be linguistically and semantically equivalent to the original MoCA, unambiguous, and easily comprehensible. The final version of MoCA-ChLA items is shown in Figure  S1, see Supplementary Materials online at doi:10.1155/2012/204623.

An instruction manual for the administration and scoring of the MoCA-ChLA was developed and used to train 15 Mandarin, Cantonese, or Taiwanese-speaking interviewers. Subtle flaws in the manual were corrected after feedback and field testing of 178 subjects. Subsequently, all interviewers underwent rigorous rater training: received a copy of the final Record Form (Figure  S1) and Manual to study, participated in two in-person training sessions, and passed a 12-item multiple-choice quiz on the administration and scoring of the MoCA-ChLA, before starting formal field testing.

### 2.3. Study Population

**Figure 1:**
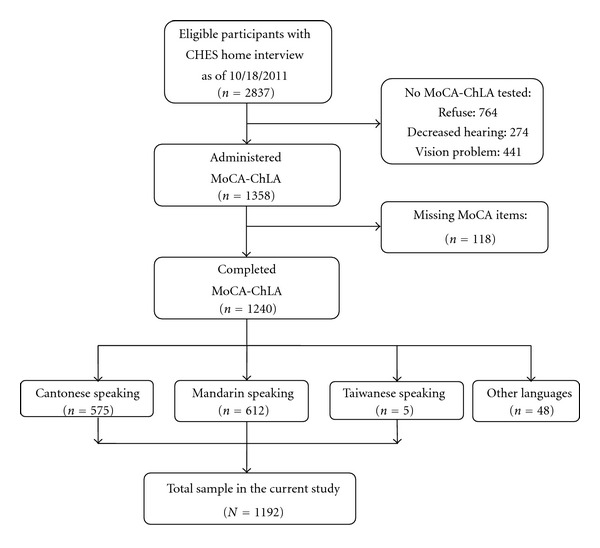
Study population.

The MoCA-ChLA was administered as part of the Chinese American Eye Study (CHES) during an in-home interview. The CHES is a population-based study designed to estimate the prevalence of visual impairment and major eye diseases among Chinese Americans aged at least 50 years residing in 9 census tracts in Monterey Park, Los Angeles County. We interviewed 2,837 eligible participants at home as of October 18, 2011 (Figure 1). Of these, 764 refused MoCA-ChLA screening, 274 could not be tested due to self-reported decreased hearing, and 441 could not be tested due to self-reported vision problems. Among 1,358 persons tested with the MoCA-ChLA, complete test forms were obtained for 1,240. All participants were tested in their preferred language; 575 were tested in Cantonese, 612 in Mandarin, 5 in Taiwanese, 4 in Vietnamese, and 44 in English. The latter two subgroups were excluded, resulting in a total of 1,192 participants in our analyses.

 We subdivided the sample by age and education. The median education of 12 years was used to divide the sample into high-(12–20 years) and low-(1–11 years) education groups. The low-education group was further subdivided into mid-low (7–11 years) and low-low (1–6 years) groups. The median age of 60 years was used to divide the sample into middle-age (50–59 years) and older age (60–100 years) groups. The older age group was further subdivided into young-old (60–74 years) and old-old (75–100 years) groups. The definition of these subgroups is somewhat arbitrary; however, these divisions allow sufficient sample size in each category and are comparable with groupings reported in the literature.

### 2.4. Statistical Analysis

The MoCA-ChLA total score equals the sum of all its subitems unadjusted for years of education. The distribution of the MoCA-ChLA total score and demographic variables was examined and nonparametric analyses were performed where appropriate. Demographic characteristics, MoCA-ChLA total score, and subitem scores were compared between the Cantonese- and Mandarin-speaking groups among the 3 education groups (1–6, 7–11, and 12–20 years) and among the 3 age groups (50–59, 60–74, 75–100 years) using Wilcoxon rank sum tests and chi-square tests. Due to very small sample size (*n* = 5), individuals who preferred to be tested in Taiwanese were not included as a separate dialect comparison group. Cronbach's coefficient alpha was used to estimate the internal consistency of the MoCA-ChLA for the total sample and for the Mandarin-speaking and the Cantonese-speaking subgroups separately.

Nonparametric analysis of covariance (rank ANCOVA) was used to compare MoCA-ChLA total score and its sub-item scores between the two dialect groups adjusting for age and education (gender was not included as a covariate as the gender distribution did not differ between the 2 dialect groups). In order to clarify the relationships between MoCA-ChLA score and age, education, and gender, rank ANCOVA was performed to compare MoCA-ChLA total score (1) among the 3 education groups controlling for age, gender, and testing dialect; (2) among the 3 age groups controlling for education, gender, and testing dialect; (3) between men and women controlling for age and education (testing language was not included as a covariate as it did not differ between men and women).

For each education group, we determined the 15th percentile of the MoCA-ChLA score in the middle-age group (MP15) to explore how age relates to possible cognitive impairment. The MP15 scores were 18 in the low-low (1–6 years), 20 in the mid-low (7–11 years), and 22 in the high-(12–20 years) education groups. The Cochran-Mantel-Haenszel test was used to test whether the percent of participants at or below MP15 score differs as a function of advancing age controlling for level of education.

Item response theory (IRT) methods [13] were used to evaluate the test characteristic curves (TCCs) and test information curves (TICs) of the MoCA ChLA total score and 6 cognitive domain scores (Table 1 shows items comprising each domain). A TCC represents a nonlinear regression of the total or domain scores on ability. It can be a very useful tool for evaluating the range of measurement and the degree of discrimination at different points of the ability continuum. In addition, the degree to which the TCC is linear provides an indication of the extent to which the measure provides interval scale or linear measurement [14]. A TIC relates latent ability in SD unit to the information (precision of measurement) for the total or domain scores. The information on the *y*-axis is the reciprocal of the variance of measurement. The TIC provides a means to ascertain what range of ability levels a test is optimally suited to measure [15]. A graded response model [16] was implemented using the R package ltm (http://www.r-project.org/). All statistical testing except IRT analysis was performed at a two-sided 5% level of significance and used Statistical Analysis System version 9.2 software (SAS Institute, Cary, NC, USA).

## 3. Results

### 3.1. Demographic Characteristics

**Figure 2:**
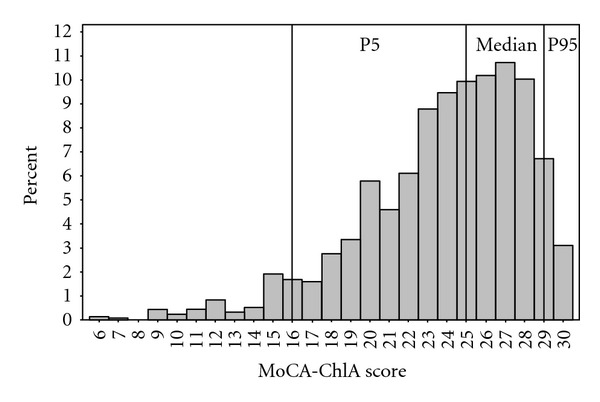
Distribution of MoCA-ChLA score (*N* = 1192) P5: the 5th percentile P95: the 95th percentile.

We administered the MoCA-ChLA to 1,192 (61.6% female) Chinese Americans living in the city of Monterey Park, Los Angeles County, California. Their mean age at testing was 62.5 years (median 60 years, range 50 to 100 years) and mean level of education was 11.6 years (median 12 years, range 1 to 20 years). Their mean MoCA-ChLA score was 23.8 (SD 4.2, median 25, range 6 to 30). Only 3% of participants received a perfect score of 30. The 5th, 50th, and 95th percentiles were 16, 25, and 29, respectively, (Figure 2).

### 3.2. Comparisons by Preferred Dialect

 Compared to Cantonese-speaking participants, Mandarin-speaking participants on average were 3 years older, had 3 more years of education, and had 1 point higher MoCA-ChLA scores (Table 2). The gender distribution did not differ between the 2 groups. After adjustment for age and education, the two dialect groups did not differ in MoCA-ChLA total and item scores, except on sentence repetition, where Mandarin speakers scored significantly better. 64.1% of Mandarin (versus 49.6% of Cantonese speakers) received a full score on sentence repetition (*P* = 0.003) (data not shown).

The Cronbach's coefficient alpha of MoCA-ChLA as an index of internal consistency was 0.79, 0.78, and 0.79, respectively, for the whole sample (*n* = 1,192) and for the Mandarin (*n* = 612) and Cantonese (*n* = 575) speakers. In the total sample, the standardized scores of Cronbach's coefficient alpha ranged from 0.77 to 0.79 for all 12 items of the MoCA-ChLA.

### 3.3. Effect of Education

Females were disproportionately represented in the low-low education group: 72%, 61%, 59% in the low-low-, mid-low-, and high-education groups, respectively (*P* = 0.01) (Table 3). The low-low education group was significantly older than the higher education groups (*P* = 0.01). Significantly more participants (75.3%) were tested in Cantonese in the low-low education group while more participants (61.9%) were tested in Mandarin in the high-education group (*P* < 0.0001). Lower education levels were associated with lower MoCA-ChLA scores after adjustment for age, gender, and testing dialect (*P* < 0.0001).

**Figure 3:**
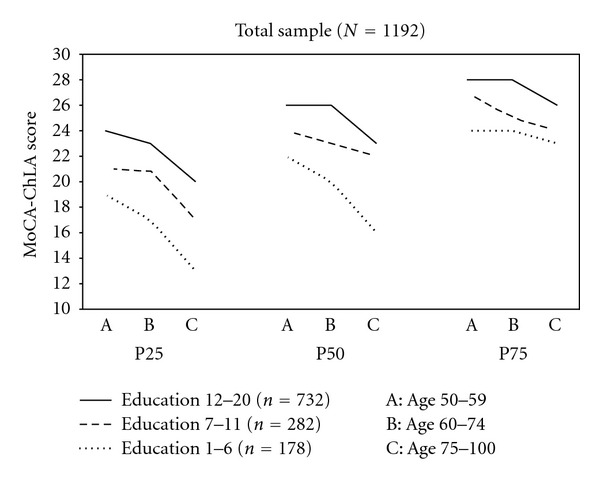
The 25th, 50th, and the 75th percentile of MoCA-ChLA score by age and education groups.

Ceiling effects were more prevalent in the high-education group. The 25th, 50th, and 75th percentiles for MoCA-ChLA scores are shown by the 3 education and the 3 age groups to mitigate ceiling effects (Figure 3). The distribution of the ranks of MoCA-ChLA score varied significantly across the 3 education groups after controlling for age (*P* < 0.0001 from the Friedman's chi-square test). A comparison between the middle-age (50–59) group and old-old (75–100) group shows that, for the P25 scores, there were approximately 4, 4, and 6 points of difference for the high-, middle-low-, and low-low-education groups. The corresponding numbers for the P50 scores were 3, 2, 6, and those for the P75 scores were 2, 3, and 1. In other words, in general age-related differences were greatest for the P25 scores, less so for the P50 scores, and least for the P75 scores. Even for the low-low-education group age-related differences in P25 and P50 score were prominent but minimal for the P75 score.

### 3.4. Effect of Age

Females were disproportionately represented in the middle-age group: 65.8%, 60.0%, and 49.6% in the middle-age, young-old, and old-old age groups, respectively (*P* = 0.001) (Table 4). Advanced age was associated with higher education with borderline significance (*P* = 0.06). Significantly more participants (70.2%) were tested in Mandarin in the old-old group than the other 2 age groups (48.5% in the middle-age group, 49.3% in the young-old group) (*P* < 0.0001). Advanced age was significantly associated with lower MoCA-ChLA scores with or without adjustment for education, gender, and testing dialect (*P* < 0.0001).

**Figure 4:**
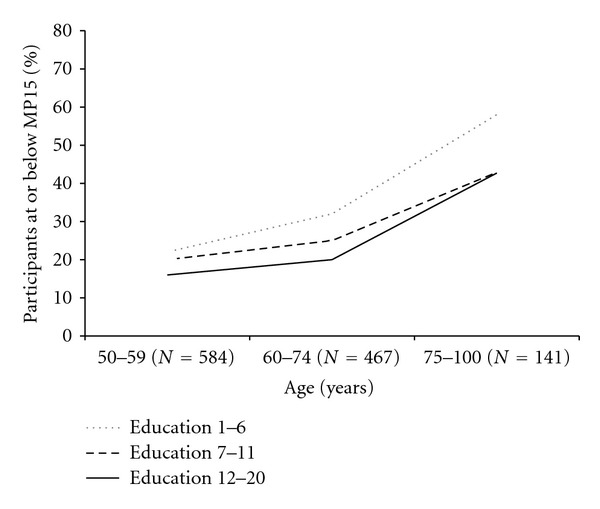
Percent of participants at or below the MP15 (the 15th percentile of the MoCA-ChLA score in the 50–59 age group).

The percentage of participants who were at or below the MP15 score increased with age, especially among the young-old and old-old groups across all three education groups (Figure 4). In each education group, multiple participants achieved the exact MP15 score. Therefore the percentage of participants who were at or below the MP15 score in each education group was greater than 15%. The association between MP15 and age remained strong after adjusting for education (*P* < 0.0001 from the Cochran-Mantel-Haenszel test).

### 3.5. Effect of Gender

Men were on average 2 years older and had 1 more year of education than women (Table 5). Fifty percent of women versus 53.5% men were tested in Mandarin (*P* = 0.39). The mean MoCA-ChLA score did not differ between men and women (*P* = 0.52). The association remained nonsignificant after adjustment for age and education (*P* = 0.59).

### 3.6. IRT Results

**Figure 5:**
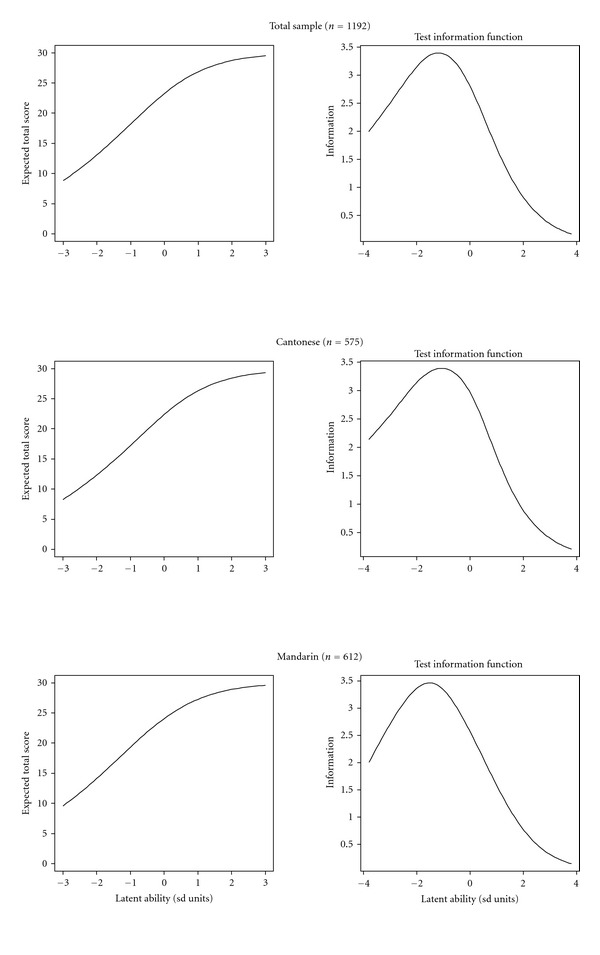
Test characteristic curves and test information curves of the MoCA ChLA total score. The upper figures are for the total sample (*n* = 1192); the middle figures are for the Cantonese speakers (*n* = 575); the lower figures are for the Mandarin speakers (*n* = 612).

TCCs for the MoCA ChLA score in the total sample, Cantonese-, and Mandarin-speaking groups are shown in Figure 5. These curves relate latent ability/cognitive function in SD units to the expected total score. The MoCA-ChLA test demonstrated similar measurement properties in the total sample and the 2 testing dialect groups. Nonlinearity was apparent for the high-functioning individuals. A 2-SD change in ability from 3 to 1 corresponded to an approximate 3 point loss in MoCA-ChLA score, while a 2-SD change in ability from −3 to −1 corresponded to an approximate 10 point loss in MoCA-ChLA score. This indicated very little floor effect and some ceiling effect of the MoCA-ChLA. The TICs relate latent ability in SD unit to the precision of measurement for domain scores. The TIC of the MoCA ChLA for the total sample peaked around −1, indicating that the MoCA ChLA mainly provides information for respondents with low to average ability (−4 to 1). Similar patterns of discrimination ability and measurement precision were observed for the 2 testing dialect groups.

**Figure 6:**
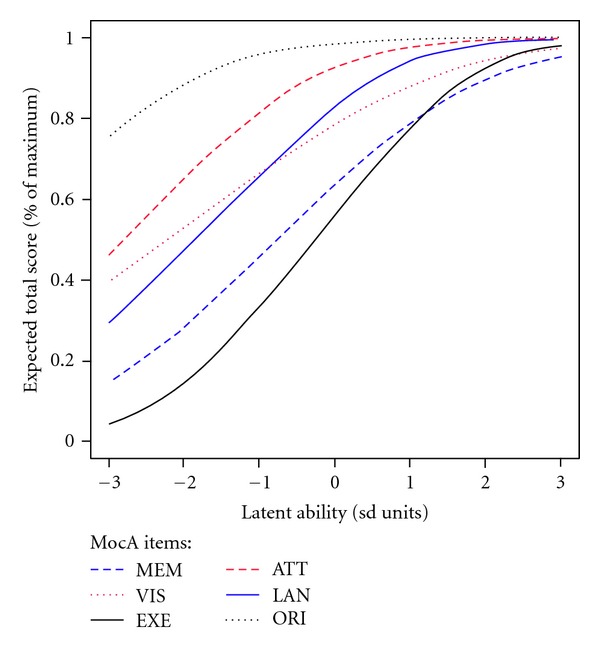
Test characteristic curves (TCCs) for the MoCA-ChLA domain scores in the total sample MEM: short-term memory; VIS: visuospatial construction; EXE: executive function; ATT: attention and concentration; LAN: language; ORI: temporal and spatial orientation.

TCCs for the 6 cognitive domain scores are shown in Figure 6. The TCCs relate latent ability in SD units to the expected total domain score (% of maximum score, for comparability across domains). All 6 domain scores showed reduced discrimination at high-ability levels. The temporal and spatial orientation domains, attention and concentration domains, and the language domain showed lower discrimination and some ceiling effect in the ability range of 1 to 3. The executive function and memory domains demonstrated high-discrimination ability for the ability range of −2 to 2. Similar patterns of domain discrimination were observed among the total sample and the 2 testing dialect groups (figures not shown).

**Figure 7:**
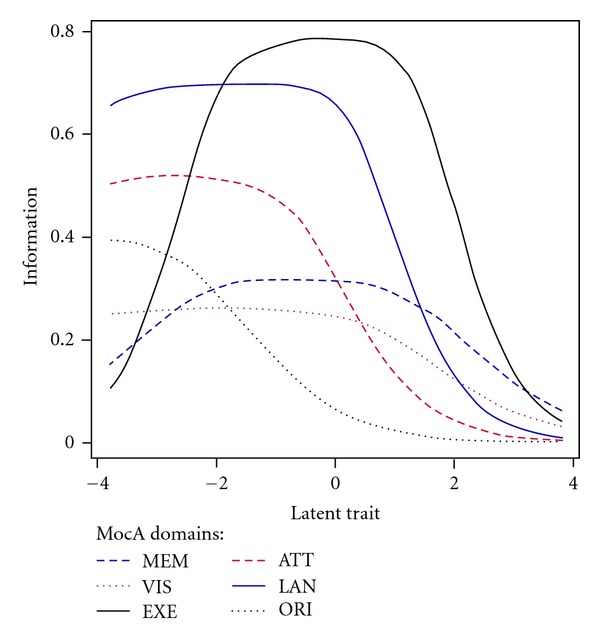
Test information curves (TICs) for the MoCA-ChLA domain scores in the total sample MEM: short-term memory; VIS: visuospatial construction; EXE: executive function; ATT: attention and concentration; LAN: language; ORI: temporal and spatial orientation.

TICs for the 6 cognitive domain scores are shown in Figure 7. The level of information provided by each of the 6 domains varied, with the executive function domain providing the highest precision. The TICs for the executive function domain and the memory domain peaked in the ability range of −2 to 2. For the language domain and the visual spatial domain, most test information was contained in the ability range of −3 to 1. The attention domain and the orientation domain provided little information for ability level of 0 or above.

## 4. Discussion

We administered the MoCA-ChLA to 1,192 (61.6% female) Chinese-speaking community residents in Monterey Park, Los Angeles County, California. The MoCa-ChLA had small ceiling effect and no floor effect and good internal consistency regardless of whether the participants spoke Mandarin or Cantonese. Similar to other cognitive screening instruments [17], the MoCA-ChLA score increased with more years of education, decreased with age, and was not related to gender. Compared to the education 1–6 years group, the mean MoCA-ChLA score was 2.6 and 4.6 higher in the education 7–11 and 12–20 years groups, respectively. We did not interview a collateral informant regarding possible dementia or cognitive decline and could not determine whether prevalence of dementia might have differed by educational group. Therefore, it would be premature to recommend adjustments in the MoCA score based on level of education, especially in the old-old groups. Compared to other cognitive domain scores, IRT analysis showed that the executive function domain demonstrated the best discriminating power and precision in measurement for participants in the cognitive function range of ±2SD. These properties support the MoCA-ChLA as a useful screening tool, with appropriate adjustments for education level, for studies of aging and dementia among individuals speaking the two major Chinese dialects of Mandarin and Cantonese.

 A major strength of our study is the large sample size of Cantonese or Mandarin speakers that were tested using one common written version MoCA-ChLA. Nunnally and Berstein suggested 0.70 as an acceptable value for the Cronbach's coefficient alpha [18]. In our study, the MoCA-ChLA total score, as well as all the subitems score, demonstrated reasonable internal consistency. This is one of the first studies to demonstrate comparability of a cognitive impairment screening tool in these two dialects. Significantly more participants (75.3%) were tested in Cantonese in the low-low-education group while more participants (61.9%) were tested in Mandarin in the high-education group (*P* < 0.0001), which may reflect historically earlier immigration of blue collar workers from Canton province in southern China. Although the Mandarin speakers scored several points higher than the Cantonese speakers, these differences were no longer significant after correcting for education.

 There may be two likely reasons for the small number (*n* = 5) of participants who expressed a preference to be tested in Taiwanese: (1) In the 20th century, the population living in Taiwan comprised 2 major groups: mainlanders who migrated from mainland China to Taiwan in 1949 and native Taiwanese. In the 1970s and 1980s, the majority of immigrants from Taiwan who settled in Monterey Park were mainlander Chinese [6, 19], whose mother language is Mandarin rather than Taiwanese dialect. (2) Since 1949, Mandarin has been the language taught in all schools and used as the official language throughout Taiwan; native Taiwanese may prefer Mandarin when taking a test even if they use Taiwanese dialect in their daily lives.

 Another strength of our study is a sizable number of participants with less than 12 years of education (*n* = 181 for 1–6 years of education, *n* = 282 for 7–11 years of education). The effects of age and education are in the expected directions, as we observed higher MoCA-ChLA scores among younger and higher educated participants. Our data indicate that education is a more potent variable than age in predicting the performance on MoCA-ChLA. After adjusting for age, the mean MoCA-ChLA scores were, respectively, 2.0 and 4.6 points lower in the mid-low-(7–11 years) and low-low-(1–6 years) education groups in comparison to that of the high-education (12–20 years) group (Table 3). Earlier studies have shown that, for cognitive screening tests, the effect of education on test score is greatest at the low end of the education spectrum [20]. Our recommendations of adding 2 points for persons with 7–11 years of education and 5 points for 1–6 years of education are significantly larger than the 1 point addition recommended in the original MoCA publication for persons with less than 12 years of education. In the latter study, the sample size was modest (*n* = 277), and the proportion of participants with less than 6 years of education was not specified. Caution should be exercised in adopting the same rule in other studies. In epidemiologic studies where a significant proportion of participants are poorly educated, the prevalence of cognitive impairment may be overestimated by following the limited education adjustment recommended by the original MoCA paper. For example, in a Korean study, more than 50% of the participants completed 6 years of education or less, but only 1 point was added to the MoCA score for this education group [8].

 We found a significant association between MoCA-ChLA and age after controlling for the level of education. Moreover, scoring at or below the 15th percentile of the middle-age group, used as a surrogate marker for cognitive impairment, was most prevalent in participants aged 75 or older with less than 7 years of formal schooling (Figure 4). The significance of these findings in this cross-sectional study is not clear and could result from several factors: (1) low-education is a vulnerability factor for normal cognitive aging, (2) low education is an independent risk factor for MCI or dementia, or (3) birth cohort effect may differentially affect the old-old group.

 Women were disproportionately represented in the low-low education group. This is not surprising, given the cultural deemphasis on education of women in China prior to the second half of the 20th century. Men were on average 2 years older and had 1 more year of education than women. Gender did not affect MoCA-ChLA score before and after controlling for age and education.

 IRT analysis suggested that the executive function domain had the best discriminating power and highest precision in measurement for participants in the cognitive function range of ±2SD. The memory domain also demonstrated high-discrimination ability although the level of precision is not as high as the executive domain. The executive function domain included the items of alternating trail making, generation of animal names, and verbal abstraction/similarities. Our finding supported the MoCA-ChLA in screening both executive and memory dysfunction in Chinese speaking Cantonese or Mandarin. These domains are particularly important when screening for cognitive impairment related to cerebrovascular and Alzheimer diseases.

 Several limitations of our study should be considered. Although the MoCA-ChLA was administered by trained interviewers, data from a collateral informant regarding the participants' cognitive, affective, and physical conditions or functional decline were not obtained. Further characterization of the sample will be needed to (1) provide normative data, (2) identify cases of MCI and dementia, (3) determine cut points for optimal sensitivity and specificity for the diagnosis of MCI, (4) refine adjustment scores for individuals with low education, and (5) verify the hypotheses regarding age-related cognitive decline.

## Supplementary Material

The MoCA Chinese Los Angeles version (MoCA-ChLA) was adapted from the Montreal Cognitive Assessment (MoCA) (Z.S. Nasreddine et al 2005) and developed as a single uniform screening tool for mild cognitive impairment among Mandarin-, Taiwanese-, and Cantonese-speaking individuals. In developing the MoCA-ChLA, we attempted to maintain the neuropsychological intent of the original MoCA instrument, as well as address the need for a linguistically and culturally appropriate cognitive screening test.
Z. S. Nasreddine, N. A. Phillips, V. Bedirian et al., “The montreal cognitive assessment, MoCA: a brief screening tool for mild cognitive impairment,” Journal of the American Geriatrics Society, vol. 53, no. 4, pp. 695–699, 2005.

## Figures and Tables

**Table 1:** MoCA-ChLA domains and subitems.

Primary domain	Test Subitem	Score range
Short-term memory	Recall 5 words after 5 min.	0–5
Visuospatial construction	Clock drawing	0–3
	Copying a cube	0-1
Executive function	Alternating trail making	0-1
	Generating animal names	0-1
	Similarities	0–2
Attention and concentration	Digit spans (forward & backward)	0–2
	Target detection	0-1
	Five serial subtractions of 7	0–3
Language	Naming 3 animals	0–3
	Repeating 2 sentences	0–2
Temporal and spatial orientation	To time and place	0–6

Total		0–30

**Table 2:** Comparisons of age, gender, education, and MoCA-ChLA scores between Cantonese and Mandarin speakers.

Characteristics	Cantonese speaking (*n* = 575)	Mandarin speaking (*n* = 612)	*P* value^1^	Adjusted *P*-value^2^
Age in years, mean ± SD	61 ± 8.5	63.9 ± 11	<0.0001	
Women, no. (%)	363 (63.1)	367 (60.0)	0.26	
Years of education, mean ± SD	10.3 ± 3.8	12.9 ± 3.7	<0.0001	
Unadjusted MoCA, mean ± SD	23.3 ± 4.2	24.4 ± 4.1	<0.0001	
Adjusted MoCA^2^, lsmean ± SE	23.7 ± 0.16	23.9 ± 0.15		0.36

^1^From Wilcoxon rank sum test for continuous variables and chi-square for gender.

^2^ *P*-value obtained from nonparametric analysis of covariance with adjustment for age and education.

**Table 3:** Comparison of age, gender, test language, and MoCA-ChLA score among the three education groups.

Variables	Education 1–6 years (*n* = 178)	Education 7–11 years (*n* = 282)	Education 12–20 years (*n* = 732)	*P* value^1^	Adjusted *P* value^2^
Age in years, mean ± SD	63.6 ± 9.7	61.1 ± 8.8	62.8 ± 10.4	0.01	
Female, no. (%)	128 (71.9)	172 (61.0)	434 (59.3)	0.01	
Testing language, no. (%)					
Cantonese	134 (75.3)	167 (59.2)	274 (37.4)	<0.0001	
Mandarin	44 (24.7)	115 (40.8)	453 (61.9)		
Taiwanese	0 (0)	0 (0)	5 (0.7)		
Unadjusted MoCA, mean ± SD	20.2 ± 4.7	23.1 ± 3.9	25.0 ± 3.6	<0.0001	
Adjusted MoCA^2^, lsmean ± SE	19.7 ± 0.6	22.3 ± 0.6	24.3 ± 0.6		<0.0001

^1^ *P* value obtained from Kruskal-Wallis test for continuous variables, from Chi-square test for gender, from Fisher exact test for testing language.

^2^ *P* value obtained from nonparametric analysis of covariance with adjustment for age, gender, and testing language.

**Table 4:** Comparison of education, gender, test language, and MoCA-ChLA score among the three age groups.

Variables	Age 50–59 years (*n* = 584)	Age 60–74 years (*n* = 467)	Age 75–100 years (*n* = 141)	*P* value^1^	Adjusted *P* value^2^
Education in years, mean ± SD	11.4 ± 3.6	11.9 ± 4.2	12 ± 4.4	0.06	
Female, no. (%)	384 (65.8)	280 (60.0)	70 (49.6)	0.001	
Testing language, no. (%)					
Cantonese	301 (51.5)	233 (49.9)	41 (29.1)	<0.0001	
Mandarin	283 (48.5)	230 (49.3)	99 (70.2)		
Taiwanese	0 (0)	4 (0.9)	1 (0.7)		
Unadjusted MoCA, mean ± SD	24.4 ± 3.8	23.9 ± 4.2	21.3 ± 5.1	<0.0001	
Adjusted MoCA^2^, lsmean ± SE	23.6 ± 0.6	22.8 ± 0.6	20.1 ± 0.6		<0.0001

^1^ *P* value obtained from Kruskal-Wallis test for continuous variables, from Chi-square test for gender, from Fisher exact test for testing language.

^2^ *P* value obtained from nonparametric analysis of covariance with adjustment for education, gender, and testing language.

**Table 5:** Comparisons of age, education, testing language, and MoCA-ChLA scores between men and women.

Characteristics	Men (*n* = 458)	Women (*n* = 734)	*P* value^1^	Adjusted *P* value^2^
Age in years, mean ± SD	63.8 ± 10.4	61.7 ± 9.6	0.0003	
Years of education, mean ± SD	12.2 ± 3.9	11.3 ± 3.9	0.0002	
Testing language, no. (%)				
Cantonese	212 (46.3)	363 (49.5)	0.39	
Mandarin	245 (53.5)	367 (50)		
Taiwanese	1 (0.2)	4 (0.5)		
Unadjusted MoCA, mean ± SD	23.9 ± 4	23.8 ± 4.3	0.52	
Adjusted MoCA^2^, lsmean ± SE	23.8 ± 0.1	23.8 ± 0.2		0.59

^1^From Wilcoxon rank sum test for continuous variables and Fisher exact test for testing language.

^2^ *P* value obtained from nonparametric analysis of covariance with adjustment for age and education.
