# Supplementary material for: Chinese-Language Montreal Cognitive Assessment for Cantonese or Mandarin Speakers: Age, Education, and Gender Effects
Source: Int J Alzheimers Dis. 2012 Jul 9;2012:204623. doi: 10.1155/2012/204623 (PMC3399373; doi:10.1155/2012/204623)
Supplement: Supplementary file 1 — The MoCA Chinese Los Angeles version (MoCA-ChLA) was adapted from the Montreal Cognitive Assessment (MoCA) (Z.S. Nasreddine et al 2005) and developed as a single uniform screening tool for mild cognitive impairment among Mandarin-, Taiwanese-, and Cantonese-speaking individuals. In developing the MoCA-ChLA, we attempted to maintain the neuropsychological intent of the original MoCA instrument, as well as address the need for a linguistically and culturally appropriate cognitive screening test. Z. S. Nasreddine, N. A. Phillips, V. Bedirian et al., “The montreal cognitive assessment, MoCA: a brief screening tool for mild cognitive impairment,” Journal of the American Geriatrics Society, vol. 53, no. 4, pp. 695–699, 2005. [file 204623.f1.pdf]

中文“蒙特利尔认知评估”洛杉矶版 CHES ID __ __ __ __-__ __ __ __-__ __ __ __-__ __ __ __

出生日期: __ __ / __ __ / 19 __ __ (月/日/年) 圈性别: 女 男 教育水平：__ __ 年

受测者惯用语: __ __ __ 施测用语: __ 1- 广东话, 2-普通话, 3-台湾话, 4- 英语, 5- 其他:

施测日期: __ __ / __ __ / 201 __ (月/日/年) 施测者: __ __ __ (拒绝回答的问题请标 99)

视空间与执行功能

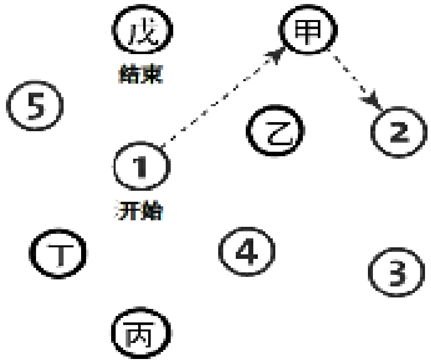

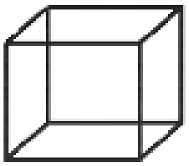

照画立方形

画时钟示时 （11 点 10 分）

轮廓 数字 指针

得分 __/5

命名

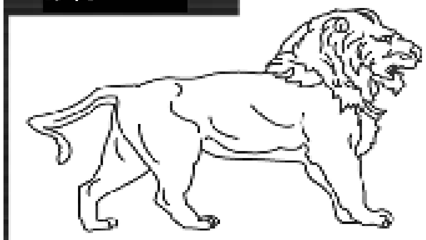

[ ]

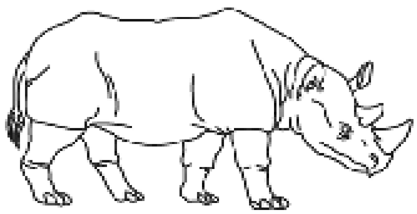

[ ]

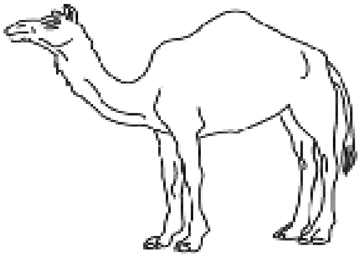

[ ]

__/3

记忆

每秒 1 词，立刻回忆，共 2 次。答对的标 ‘1’，否则标 ‘0’。告知将再测。

	头发	茶杯	教堂	菊花	红色
第一次					
第二次					

注意

每秒 1 字。记下回答。 （顺背）：2－1－8－5－4 答：2－1－8－5－4 [ ]
（倒背）：7－4－2 答：2－4－7 [ ] __/2

每秒 1 字。听到敲桌时，在相应的数字上画圈。
5-2-1-3-9-4-1-1-8-0-6-2-1-5-1-9-4-5-1-1-1-4-1-9-0-5-1-1-2 __/1

100 连续减 7，记下答案（最多 5 个）：[ ] [ ] [ ] [ ] [ ] __/3

语言

重复(全对的标 ‘1’，否则标 ‘0’)： 我只知道 今天 该小张来帮忙 （6 秒） [ ]
狗在房间的时候，猫总是躲在沙发下面（6 秒） [ ] __/2

四支脚动物的名字 ： 30 秒|30 秒： [ ] | [ ] __/1

抽象

香蕉-桔子=水果 火车-自行车/ 脚踏车/ 单车 [ ] 手表-尺 [ ] __/2

延迟回忆

	头发	茶杯	教堂	菊花	红色
未提示					
类别提示					
三项选一					

__/5

定向

年 [ ] 月 [ ] 日 [ ] 星期几 [ ] 街 [ ] 城市 [ ] __/6

分数极可能无效，详见背面：______

Figure1. The MoCA-ChLA Record Form
